# Monitoring the Chemical Profile in Agarwood Formation within One Year and Speculating on the Biosynthesis of 2-(2-Phenylethyl)Chromones

**DOI:** 10.3390/molecules23061261

**Published:** 2018-05-25

**Authors:** Ge Liao, Wen-Hua Dong, Jin-Ling Yang, Wei Li, Jun Wang, Wen-Li Mei, Hao-Fu Dai

**Affiliations:** 1 Key Laboratory of Biology and Genetic Resources of Tropical Crops, Ministry of Agriculture, Institute of Tropical Bioscience and Biotechnology, Chinese Academy of Tropical Agricultural Sciences, Haikou 571101, China; liaoge828@aliyun.com (G.L.); dongwenhua@itbb.org.cn (W.-H.D.); jinlyang@126.com (J.-L.Y.); liwei@itbb.org.cn (W.L.); wangjun@itbb.org.cn (J.W.); 2 College of Horticulture and Landscape Architecture, Hainan University, Haikou 570228, China; 3 Hainan Engineering Research Center of Agarwood, Haikou 571101, China

**Keywords:** Agarwood, *Aquilaria sinensis*, 2-(2-Phenylethyl)chromones, biosynthesis

## Abstract

Agarwood is highly valued for its uses as incense, perfume, and medicine. However, systematic analyses of dynamic changes of secondary metabolites during the process of agarwood formation have not yet been reported. In this study, agarwood was produced by transfusing the agarwood inducer into the trunk of *Aquilaria sinensis*, and changing patterns of chemical constituents, especially 2-(2-phenylethyl)chromones (PECs), in wood samples collected from the 1st to 12th month, were analyzed by GC-EI-MS and UPLC-ESI-MS/MS methods. Aromatic compounds, steroids, fatty acids/esters, sesquiterpenoids, and PECs were detected by GC-MS, in which PECs were the major constituents. Following this, UPLC-MS was used for further comprehensive analysis of PECs, from which we found that 2-(2-phenylethyl)chromones of flindersia type (FTPECs) were the most abundant, while PECs with epoxidated chromone moiety were detected with limited numbers and relatively low content. Speculation on the formation of major FTPECs was fully elucidated in our context. The key step of FTPECs biosynthesis is possibly catalyzed by type III polyketide synthases (PKSs) which condensate dihydro-cinnamoyl-CoA analogues and malonyl-CoA with 2-hydroxy-benzoyl-CoA to produce 2-(2-phenyethyl)chromone scaffold, or with 2,5-dihydroxybenzoyl-CoA to form FTPECS with 6-hydroxy group, which may serve as precursors for further reactions catalyzed by hydroxylase or *O*-methyltransferase (OMT) to produce FTPECs with diverse substitution patterns. It is the first report that systematically analyzed dynamic changes of secondary metabolites during the process of agarwood formation and fully discussed the biosynthetic pathway of PECs.

## 1. Introduction

Agarwood is the fragrant resinous heartwood from trees of the genus *Aquilaria*. It is widely accepted that the precious and high-priced agarwood has been of great significance in Asian cultures for centuries. For example, it has been used as incense in Buddhist, Hindu, and Islamic ceremonies, and also as traditional medicine in Ayurveda and Chinese therapies [1]. Healthy *Aquilaria* trees, however, hardly contain resin unless they were stimulated by various forms of injury or microbial attack [2,3,4,5]. Agarwood-producing plants are timber species which take quite a long time to grow and the agarwood-formation process is also time consuming. This results in the fact that wild agarwood is extremely rare and cannot satisfy the sustainable market need for wild agarwood.

Under certain circumstances, great efforts are under way to produce agarwood by intentional injuries. The first trial initiated by Tunstall was to inoculate trees with fungi isolated from agarwood in 1929 [1]. After that, a series of progresses have been achieved in developing artificial methods of agarwood production such as fungi inoculation, physical wounding, and chemical treatment (e.g., solutions of FeCl_3_ or acetic acid) [6,7,8]. Nowadays, experienced farmers in Southeast Asia obtain agarwood by using traditional methods, such as axe chopping, nailing, holing, burning, and the partial trunk pruning method, among others.

Although a series of methods aimed at generating agarwood artificially have been developed rapidly and promoted in many plantations of *Aquilaria* species, the mechanism of agarwood formation has not been fully understood, and no convincing standard method has been established for quality control of the artificial agarwood. As far as we know, only a few studies concerned about the changes happening to *Aquilaria* trees after stimulation, e.g., investigation on post-infection changes in sugars, ascorbic acid, phenols, and protein during pathogenesis in *A. malaccensis*, showed that those primary metabolites decreased whereas secondary metabolites increased [9]. Observation of a sequential change in the wood coloration around injury sites revealed that a pale discoloration occurred after 1 month, followed by a darker yellow-brown discoloration after 3 months, which then became dark brown within 8–10 months and changed to black within 20 months and was accompanied by a burning scent [5]. Analyzing agarwood samples after induction of 15th, 30th, and 60th day, respectively, by using GC-MS showed that sesquiterpenes were detected after 6 days and the relative content of sesquiterpenes and aromatic compounds were increased with prolonged time [10].

Unlike previous studies, agarwood samples used in this study were obtained from a relatively new and efficient transfusion method with the agarwood inducer invented by our group [11]. The chemical constituents were monitored for a fixed period by using GC-EI-MS and UPLC-ESI-MS/MS methods for the purpose of revealing the dynamic changes of the chemical profile in the process of agarwood formation. The detected compounds were identified by searching established database like NIST14 and WILEY275, or matching with reference compounds obtained by our previous study to enable us to conduct qualitative analysis of 2-(2-phenylethyl)chromones (PECs) in agarwood samples by using UPLC-MS. Based on the comprehensive comparison of PECs of different origin, as well as by detailed analyses of the obtained results in this study, we firstly proposed the hypothetical biosynthesis of PECs, which provides the basis for further studies on the mechanism of agarwood formation.

## 2. Results and Discussion

### 2.1. The Results of GC-MS Analysis

The ether extracts of injured *A. sinensis*, which did not cover all the components contained in the samples, were analyzed by GC-MS leading to the characterization of 52 compounds in eight samples collected monthly from the 1st to 6th, then the 9th and 12th months, respectively (Table 1). The identification of 2-(2-phenylethyl)chromone derivatives were mainly based on their MS characterization and fragmentation patterns proposed by our group [12]. Other compounds were characterized by matching with the NIST14 and WILEY275 database and by comparing their MS spectra with those of literature data [1]. The relative contents of the compounds were determined by normalization.

These 52 compounds were classified into five types according to their structures, and the number and relative content of each type were counted and listed in Table 1. Healthy *A. sinensis* mainly contained fatty acids, alkanes, and aromatic compounds, while GC-MS analysis of ether extracts of injured *A. sinensis* revealed the appearance of 2-(2-phenylethyl)chromones, sesquiterpenoids, steroids, fatty acids/esters, and aromatic compounds in the volatile constituents, in which 2-(2-phenylethyl)chromones were the major components, existing in large amounts in the sample of the 1st month after the injury, and their relative content still accounted for more than half of the total relative content in the sample of the 12th month. As one of the characteristic constituents in agarwood, sesquiterpenoids emerged relatively late, accompanied by much lower relative content. The reason why the relative content of sesquiterpenoid was not so high was probably because the wide variety of sesquiterpenoid skeletons made them difficult to be identified, or probably because the formation time for the samples obtained in this study was not long enough for the accumulation of sesquiterpenoids.

Besides, unlike the results of previous GC-MS analysis of agarwood [13], there were some steroids detected in this study. This was probably because the column we used has the capability of high-temperature resistance, which enabled the volatilization and identification of steroids under the oven temperature of 310 °C for 10 min. Among those known compounds, clionasterol and stigmasterol as the major components of steroids in agarwood and also commonly distributed in other plants. Oleic acid and palmitic acid were the main constituents of the fatty acids, which could be the remaining compounds produced before agarwood formation [14,15]. It is noteworthy that some aromatic compounds were also detected, such as phenylpropionic acid, phenylpropionic acid methyl ester, and 4-methoxy-phenylpropionic acid methyl ester, which possibly have some relationship with the aromatic substrates of PECs biosynthesis, as well as 4-phenylbutan-2-one (benzylacetone), 4-(4-methoxyphenyl)butan-2-one (anisylacetone), and 4-(4-hydroxy-3-methoxy phenyl)butan-2-one (zingerone), which were frequently found in volatile oil of agarwood [1,13].

In conclusion, it was revealed by GC-MS analysis that sesquiterpenoids appeared relatively late and presented quite low relative content in the early stage of agarwood formation after transfusing the agarwood inducer to *A. sinensis*. On the contrary, the woods around the hole have produced plenty of PECs in the first month (total relative content was 83.86%), and among them, 6,7-dimethoxy-2-(2-phenylethyl)chromone was the dominant one with a relative content of 43.43%, which almost took up half of the total relative content of the sample in the first month. However, GC-MS is only suitable for volatile constituents and the identification via GC-MS can only be achieved within limited library searching. Therefore, given the fact that PECs compromised a high percentage of the identified compounds, while some nonvolatile PECs perhaps failed to be detected by GC-MS, UPLC-MS analysis was carried out to study the dynamic changes of PECs during the process of agarwood formation.

### 2.2. The Results of UPLC-MS Analysis

**Figure 1:**
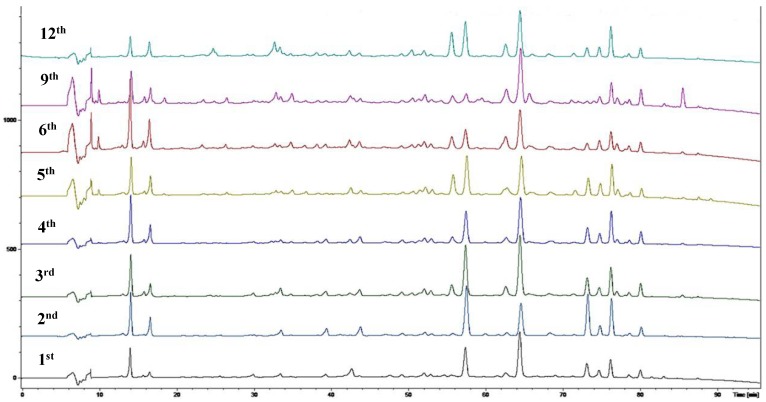
The UPLC spectrum of agarwood samples produced from the 1st to 12th months.

**Figure 2:**
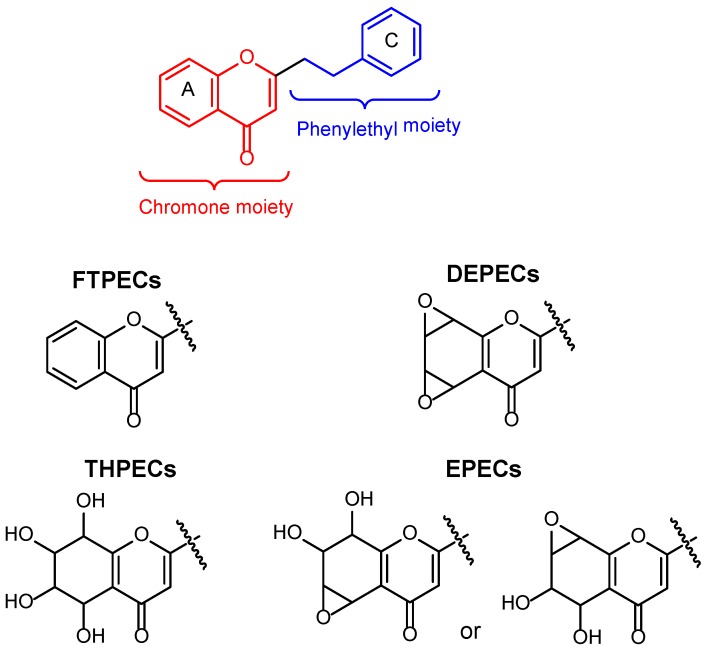
The characteristic structures of four types of 2-(2-phenylethyl)chromones.

Sixty-four 2-(2-phenylethyl)chromones (PECs) in total were detected by UPLC-MS from the obtained samples (Figure 1, Table 2). Among them, 30 compounds were identified by comparing their MS spectra with those of reference compounds (Table 3) and the structures of others were deduced on the basis of their MS characterization, fragmentations patterns, and characteristic fragment peaks. According to the characteristic structure of chromone skeleton, 2-(2-phenylethyl)chromones (PECs) can be subdivided into four groups, i.e., 5,6,7,8-tetrahydro-2-(2-phenylethyl)chromones with two epoxys linked at the cyclohexane ring shortened as DEPECs, 5,6,7,8-tetrahydro-2-(2-phenylethyl)chromones with single epoxy at the cyclohexane ring named as EPECs, 5,6,7,8-tetrahydro-2-(2-phenylethyl)chromones which possess a highly oxygenated chromone skeleton are called THPECs, and 2-(2-phenylethyl)chromones of flindersia type were abbreviated to FTPECs (Figure 2). All of the 64 compounds detected in the collected samples were classified into the above four types, and statistical analysis of the number and total relative content of each group led us to know that FTPECs were the primary group with numerous diversities and highest relative content and THPECs as the next group, while EPECs and DEPECs had limited numbers and comparatively low content (Table 2). To the best of our knowledge, DEPECs and EPECs were quite rare in nature, and such nonaromatic chromone skeletons have only been identified in agarwood [16,17,18,19,20], and no other known chromones were found to have such an epoxy group in the chromone moiety except PECs. Also, no other plants except *Aquilaria* species has been reported to contain DEPECs and EPECs.

#### 2.2.1. Discussion of Substitution Pattern of Chromone and Phenylethyl Moiety of FTPECs, Respectively

**Figure 3:**
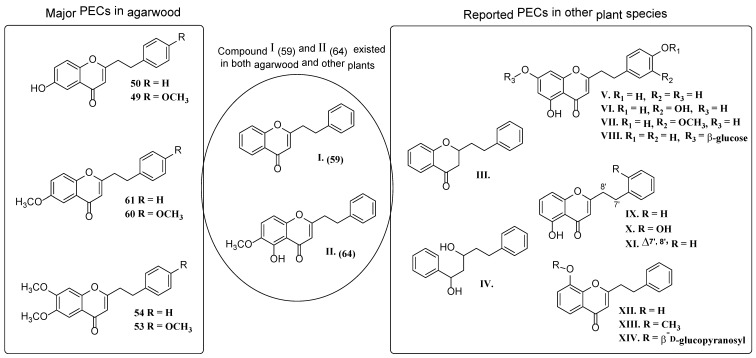
Comparison of major PECs derived from agarwood and other plant species.

Although studies on chemical constituents of agarwood did not began until 1978, the uncommon structure of 2-(2-phenylethyl)chromones intrigued many research groups, which led to the rapid exploitation of PECs. Until now, more than 100 PECs have been isolated and identified from agarwood of different origin. Numerous PECs were differentiated from each other by different substituents or substituted position. Substituents attached at PECs are commonly hydroxy and methoxy, and chlorinated PECs and glycosylated PECs also existed in agarwood but with relatively rare frequency. The substituents were most frequently linked at C6-only or both C6- and C7-position of FTPECs at chromone moiety, while C4′-only or both C3′- and C4′-position of PECs at phenylethyl moiety. The basic structure of FTPECs is a chromone skeleton bearing an uncommon phenylethyl group at the C-2 position, thus distinguishing FTPECs from other normal chromones (Figure 2). However, unlike nonaromatic PECs, FTPECs are not unique in agarwood, and a few members of FTPECs, as concluded in Figure 3, have also been identified in other plant species such as *Flindersia laevicarpa*, *Bothriochloa ischaemum*, *Imperata cylindrica*, and *Cucumis melo* (Table 4) [21,22,23,24,25,26]. Although either FTPECs from agarwood or from other plants all share the same skeleton, there were differences in substitution patterns of their chromone core. Among all FTPECs in agarwood, those isolated most frequently or existed with comparatively higher relative content were basically FTPECs with a nonsubstituted A-ring, or FTPECs with 6-hydroxy, or 6-methoxy, or 6,7-dimethoxy at chromone moiety (Figure 3), while several FTPECs isolated from other plant species were rarely found in agarwood, such as FTPECs bearing 5,7-dihydroxy isolated from *C. melo* or FTPECs bearing 5-hydroxy isolated from *I. cylindrica* or *B. ischuemurn* (Figure 3). The different substitution patterns between FTPECs of different origin suggested their different biosynthetic pathway.

**Figure 4:**
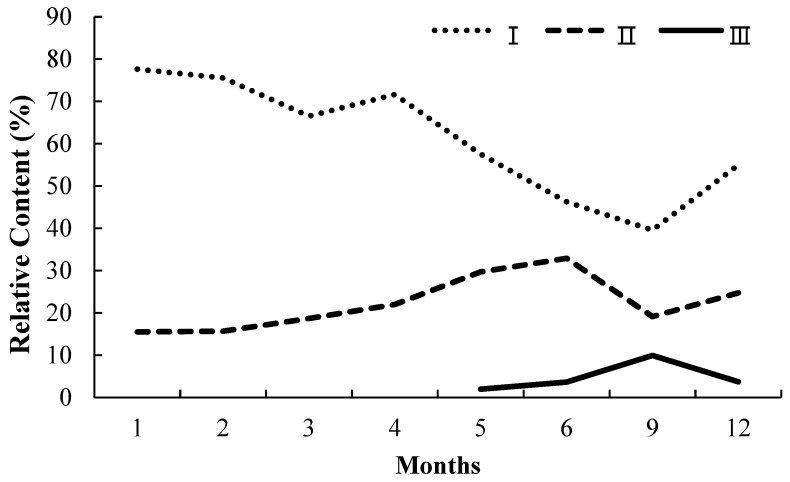
The changing trend of the total relative content from the 1st to 12th month of three groups of PECs which were subdivided according to the substitution pattern of phenylethyl moiety. Group I were PECs with no substitution at phenylethyl moiety, group II were PECs with 4′-OCH_3_ at phenylethyl moiety, group III were PECs with 3′-OH and 4′-OCH_3_ at phenylethyl moiety.

As for the substitution pattern at phenylethyl moiety of PECs from agarwood, most of them frequently appeared as a nonsubstituted C-ring, or 4′-OCH_3_-only, or both 3′-OH- and 4′-OCH_3_-substituted C-ring. As shown in Figure 4 and Table 5, from the 1st to 12th month, the total relative content of PECs with nonsubstitution at phenylethyl moiety was generally much higher than PECs with 4′-OCH_3_ at phenylethyl moiety, and the latter was also much higher than PECs with 3′-OH and 4′-OCH_3_ at phenylethyl moiety. The general trend of relative contents of PECs with a nonsubstituted C-ring was decreased gradually, for more and more PECs with diverse substituted C-rings appeared from the 1st to 12th month. The general tendency of relative content of PECs with a 4′-OCH_3_-substituted C-ring was increased gradually from the 1st to 6th month but dropped down between the 6th and 9th month, and then went up again from the 9th to 12th month, while PECs with a 3′-OH- and 4′-OCH_3_-substituted C-ring appeared only from the 5th month and showed an uptrend from the 6th to 9th month, and then decreased from the 9th to 12th month. The abovementioned results suggested that PECs with 3′-OH and 4′-OCH_3_ are hydroxylated products from 4′-OCH_3_-substituted PECs. Apart from above typical substitution pattern for phenylethyl moiety, there were also FTPECs detected in some samples bearing 3′-OCH_3_ and 4′-OH at phenylethyl moiety such as 2-[2-(4-hydroxy-3-methoxyphenyl)ethyl]chromone (**40**), 4′-hydroxylated FTPECs such as 6,7-dimethoxy-2-[2-(4-hydroxyphenyl)ethyl]chromone (**33**), and 6-hydroxy-7-methoxy-2-[2-(4-hydroxyphenyl)ethyl]chromone (**19**), as well as 2′-hydroxylated FTPECs such as 6-hydroxy-2-[2-(2-hydroxyphenyl)ethyl]chromone (**34**) and 2-[2-(2-hydroxyphenyl)ethyl] chromone (**47**) (Table 2).

#### 2.2.2. Discussion of Possible Biosynthetic Pathway of 2-(2-Phenylethyl)Chromones

**Figure 5:**
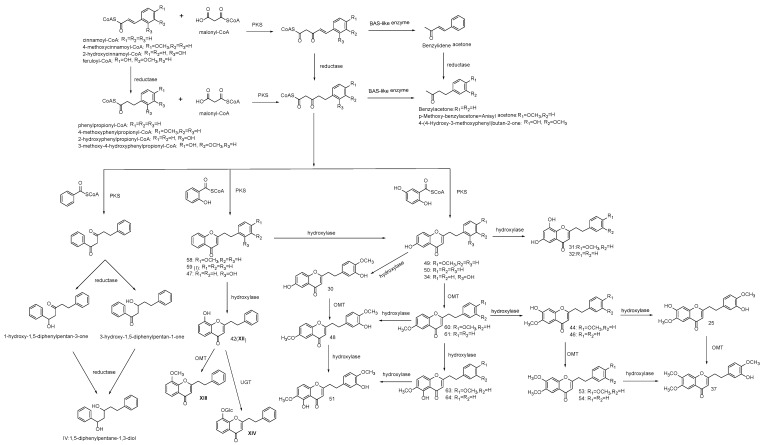
Hypothetical scheme for the biosynthetic pathway of PECs. OMT, O-methyltransferase, UGT, glycosyltransferase.

PECs are principal components responsible for the quality of agarwood. However, the molecular basis of PECs biosynthesis remains almost unknown. The most recent progress is about a new type III polyketide synthase (PKS), AsPKS1, which is isolated and characterized in *A. sinensis* calli [27], and may contribute to the biosynthesis of PECs. Type III PKSs are a group of enzymes traditionally existing in plants to produce divergent natural polyketide scaffolds, such as flavonoids, stilbenes, phloroglucinol, curcuminoids, and so on. The enzyme assay experiment of AsPKS1 carried out in vitro suggested it not only exhibited the same function as curcumin synthase (CURS) or benzalacetone synthase (BAS) to yield curcuminoids or benzylacetone analogues, but also showed the ability to condensate 4-coumaroyl-CoA with malonyl-CoA and benzoyl-CoA to produce 5-(4-hydroxyphenyl)-1-phenyl-4-pentene-1,3-dione, or condensate dihydro-4-coumaroyl-CoA with malonyl-CoA and benzoyl-CoA to produce 5-(4-hydroxyphenyl)-1-phenylpentane-1,3-dione [28]. Thus, it is possible that some PKS similar to AsPKS1 may also condensate dihydro-cinnamoyl-CoA with malonyl-CoA and benzoyl-CoA to produce 1,5-diphenylpentan-1,3-dione, which possesses the same C_6_–C_5_–C_6_ scaffold as products of AsPKS1 (Figure 5). Although 1,5-diphenylpentan-1,3-dione has not yet been reported from nature, one of its analogues, 1-hydroxy-1,5-diphenylpentan-3-one, has been found from agarwood, and another, 3-hydroxy-1,5-diphenylpentan-1-one, has been found from the medicinal plant *Daphne acutiloba* Rehd. (Thymelaeceae) [29], which possess obvious structural similarities with PECs except for a γ-pyrone ring. Referring to the chemoenzymatic results of HsPKS3 carried out in vitro by Wang et.al [30], coincubation of HsPKS3 with 2-hydroxybenzoyl-CoA and 3-oxo-5-phenylpentanoic acid led to the formation of a small amount of 2-(2-phenylethyl)chromone, while condensation of benzoyl-CoA, malonyl-CoA and *p*-hydroxyphenylpropinoyl-CoA only produced a diarylpentanoid, which suggests a hydroxy group at the C-2 position may be a prerequisite for the ring closure of the diarylpentanoid scaffold. Therefore, the formation of 2-(2-phenylethyl)chromone in vivo was possibly catalyzed by PKSs that condensate 2-hydroxybenzoyl-CoA, malonyl-CoA, and phenylpropinoyl-CoA, while 1,5-diphenylpentan-1,3-dione analogues were produced by the condensation of benzoyl-CoA with malonyl-CoA and phenylpropinoyl-CoA.

As the biosynthesis of the nonsubstituted FTPEC, 2-(2-phenylethyl)chromone (**59**), has been described as above, from which we could know that the substitution pattern at phenylethyl moiety of PECs is in accordance with the “first” starter, phenylpropinoyl-CoA, while the nonsubstituted A-ring is derived from the “second” starter 2-hydroxybenzoyl-CoA, and malonyl-CoA acts as an unchangeable “bridge” to connect the above two parts together. Thus, the biosynthesis pathway of FTPECs with diverse substitution patterns could be deduced in a similar way, that is, the chromone moiety of FTPECs was derived from the 2-hydroxybenzoyl-CoA analogues, while the phenylethyl moiety came from the phenylpropinoyl-CoA analogues (Figure 5).

When considering the substitution patterns of the chromone core of FTPECs, as mentioned before, those isolated most frequently or existed with comparatively higher relative content in agarwood were basically FTPECs with a nonsubstituted A-ring, or FTPECs with a 6-hydroxy-, 6-methoxy-, or 6,7-dimethoxy-substituted chromone moiety. According to the aforementioned biosynthetic pathway of PECs with a nonsubstituted A-ring, which was derived from 2-hydroxybenzoyl-CoA substrate, 6-hydroxy-substituted FTPECs were consequently derived from 2,5-dihydroxybenzoyl-CoA. At the same time, FTPECs with a substituted A-ring, including 6-hydroxy-substituted FTPECs, also may be modified products of FTPECs with a nonsubstituted A-ring by further reaction catalyzed by hydroxylase or *O*-methyltransferase (OMT) (Figure 5). It is difficult to deduce whether both of the two branches of pathway exist or if one of them has an advantage over the other. However, we could get some clues by considering the formation time of the identified FTPECs at least. Among all the A-ring-substituted PECs identified in this study, most of the C6-substituted PECs existed from the 1st to the 12th month with a relatively high amount, such as compounds **49** and **50** with 6-hydroxy, compounds **60** and **61** with 6-methoxy, compounds **53** and **54** with 6,7-dimethoxy, or compounds **63** and **64** with 5-hydroxy, 6-methoxy groups. Therefore, it is most likely that 2,5-dihydroxybenzoyl-CoA was involved in the biosynthetic pathway of FTPECs and produced 6-hydroxy substituted FTPECs as a precursor for further modifications. For example, 6-hydroxy-2-[2-(4-methoxyphenyl)ethyl]chromone (**49**) and 6-methoxy-2-[2-(4-methoxyphenyl)ethyl] chromone (**60**) were detected from the 1st to the 12th month, while their possible hydroxylated products, 6,8-dihydroxy-2-[2-(4-methoxyphenyl)ethyl]chromone (**31**) and 7-hydroxy-6-methoxy-2-[2-(4-methoxy phenyl)ethyl]chromone (**44**), were only detected from the 4th to the 12th month. The relatively late time formation of **31** and **44** supported that they were hydroxylated products of **49** and **60**, respectively, rather than directly formed by condensation reactions with other potential substrates beside 2-hydroxybenzoyl-CoA and 2,5-dihydroxybenzoyl-CoA. However, this study did not cover whole range of chromone compounds occurring in agarwood formation, but only the major PECs. Thus, it cannot be excluded that some other benzoyl CoA analogues may also be found to contribute to the construction of chromone moiety of FTPECs as potential substrates in further research.

As mentioned before, most of the substitution patterns at the C-ring were nonsubstituted, 4′-OCH_3_-only, or 3′-OH- and 4′-OCH_3_-substituted, and in rare cases, also 2′-OH-substituted or 3′-OCH_3_- and 4′-OH-substituted, which suggests the first starter substrates of PECs biosynthesis are most likely to correspond with 2,3-dihydro derivatives of cinnamoyl-CoA and 4-methoxycinnamoyl-CoA, and sometimes also feruloyl-CoA and 2′-hydroxycinnamoyl-CoA. Utilization of above aromatic substrates as starters was proven by the existence of their possible BAS-like enzyme catalyzed products, e.g., (*E*)-4-phenylbut-3-en-2-one (benzylidene acetone), 4-phenylbutan-2-one (benzylacetone), 1-(4-methoxyphenyl)propan-2-one (anisylacetone), as well as 4-(4-hydroxy-3-methoxyphenyl) butan-2-one in agarwood [1]. Further, the latter three compounds have also been detected in our volatile constituents produced in agarwood samples from the 2nd to the 12th month (Table 1). BAS is a plant-specific type III polyketide synthase (PKS) that has been isolated from raspberries and *Rheum palmatum* and catalyzes a one-step decarboxylative condensation of 4-coumaroyl-CoA with malonyl-CoA to produce the C_6_–C_4_ skeleton of phenylbutanoids [31,32,33]. However, apart from the substrates mentioned above, it cannot be excluded that 4-coumaroyl-CoA, caffeoyl-CoA, or other potential substrates may contribute to the biosynthesis of PECs. Those PECs bearing 3′-OH and 4′-OCH_3_ at phenylethyl moiety are most likely to be 3′-hydroxylated products from 4′-OCH_3_-substituted PECs, for the reason that the corresponding C_6_–C_3_ substrates bearing 3′-OH and 4′-OCH_3_ groups have not been found in plants, and 3′-OH and 4′-OCH_3_ substituted PECs were not detected from the beginning but have only been found since the 5th month or even later. Here, we took compounds **49**, **50**, and **30** as an example for detailed elucidation. Both 6-hydroxy-2-(2-phenylethyl)chromone) (**50**) and 6-hydroxy-2-[2-(4-methoxyphenyl) ethyl]chromone (**49**) were detected from the 1st to the 12th month, while 6-hydroxy-2[2-(3-hdroxy-4-methoxyphenyl)ethyl]chromone (**30**) only has been detected since the 5th month. As previously discussed, the phenylethyl moiety of compound **50** was suggested to derive from dihydro-cinnamoyl-CoA, and the 4-methoxylpenylethyl part of compound **49** was came from 4-methoxyphenylpropionyl-CoA. Thus, it can be explained that FTPECs with a nonsubstituted or 4-OCH_3_-substituted C-ring were produced by the condensation reactions with their corresponding substrates, while the late formation time for **30** or other 3′-OH- and 4′-OCH_3_-substituted PECs, such as **25**, **37**, **48**, and **51**, were for the reason that they were not directly formed by PKS but 3′-hydroxylated products from 4′-OCH_3_ substituted PECs.

**Figure 6:**
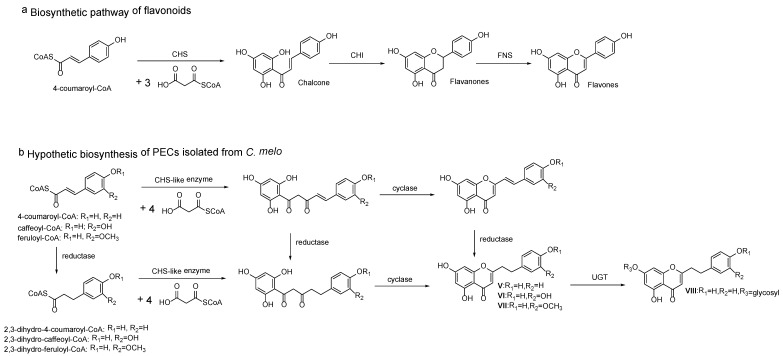
Hypothetic biosynthesis of PECs isolated from *C. melo* and comparison of the biosynthesis of flavonoids.

The possible biosynthesis pathway of major FTPECs has been elucidated in Figure 5, and other known PECs which were not detected or identified in this study, either from agarwood origin or from other plants, may also be produced by a similar biosynthesis pathway, except for FTPECs bearing 5,7-dihydroxyl groups. So far, 5,7-dihydroxylated FTPECs have only been isolated from *C. melo*, and their biosynthetic pathway may be partly different from above mentioned agarwood-type PECs. As we know, flavonoids with 5,7-dihydroxyl or other 5,7-dioxygen groups commonly exist in the stem of healthy *A. sinensis* plants, while only one flavone, 5-hydroxy-7,4′-dimethoxyflavone, has been reported in agarwood induced by artificial holing [34]. Instead, FTPECs with diverse substitution patterns other than 5,7-dihydroxylated FTPECs emerged in a large number and accumulated gradually during agarwood formation. It is known that the typical biosynthesis of flavonoids produces chromones with 5,7-dihydroxyl groups, and their biosynthetic pathway has been well-studied and elucidated as the involvement of CHS superfamily, which typically select 4-coumaroyl-CoA as a starter and perform sequential condensations with three C2 units derived from malonyl-CoA to produce an enzyme-bound tetraketide intermediate, following with Claisen-type cyclization to form a C_6_–C_3_–C_6_ scaffold of narigenin chalcone [33,35] (Figure 6). It is reasonable to deduce that the biosynthesis of FTPECs with 5,7-dihydroxylated chromone moiety may be different from agarwood-type FTPECs, the A-ring of which is derived from 2-hydroxy-benzoyl-CoA substrates and its analogues, but similar to typical flavonoids with 5,7-dihydroxyl groups. Thus, we proposed another biosynthetic pathway for 5,7-dihydroxylated FTPECs (V–VIII), which is isolated from *C. melo* as similar to the flavonoids biosynthetic pathway, but sequentially condensing four C2 units from malonyl-CoA to form a C_6_–C_5_–C_6_ scaffold of chalcone analogue, following cyclization of the linear C_6_–C_5_–C_6_ intermediate to yield 5,7-dihydroxylated FTPECs, which may serve as skeletons for further modifications (Figure 6). For the reason that the substitution pattern at phenyethyl moiety of PECs is in accordance with the starter units, the starters of the flavonoids-like pathway are proposed to be 4-coumaroyl-CoA, caffeoyl-CoA, and feruloyl-CoA, or their 2,3-dihydro derivatives, according to the structures of compounds V–VIII, which all bear 4′-hydroxy group. It is noteworthy that the substrates involved in this biosynthetic pathway are different from those substrates of agarwood-type PECs except for feruloyl-CoA.

**Figure 7:**
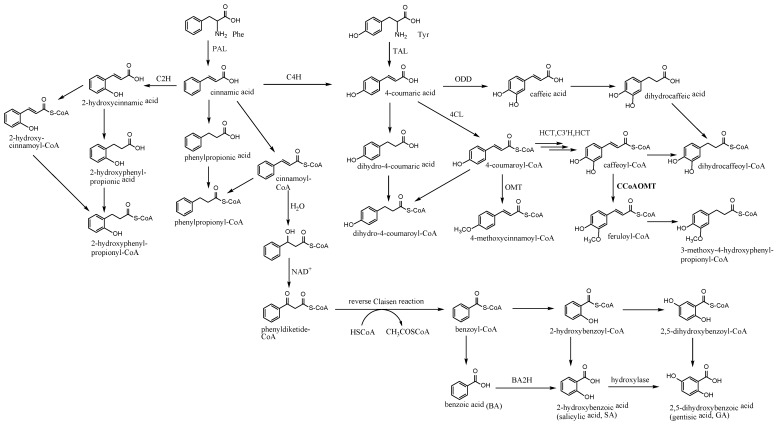
Plant phenolics based on phenylpropanoid pathway and their production as substrates for biosynthesis of polyketide class. PAL, phenylalanine ammonia lyase (MIO); TAL, tyrosine ammonia lyase; C4H, cinnamate-4-hydroxylase (O_2_, cytochrome P450, NADPH); C2H, cinnamate-2-hydroxylase (O_2_, cytochrome P450, NADPH); 4CL, 4-hydroxycinnamoyl-CoA ligase (ATP, coenzyme A [CoASH]); HCT, hydroxycinnamoyl-CoA:shikimate hydroxycinnamoyl transferase (shikimate); C3′H, *p*-coumaroyl-5-*O*-shikimate 3′-hydroxylase (O_2_, cytochrome P450, NADPH); CCoAOMT, caffeoyl-CoA *O*-methyl transferase (AdoMet); ODD, 2-oxoglutarate-dependent dioxygenase (α-ketoglutarate, Fe(II), ascorbate), BA2H, benzoic acid-2-hydroxylase.

Phenylpropanoid derived metabolites comprise and contribute to multiple biosynthetic branches other than lignin and flavonoid biosynthesis only [36]. The origin of numerous CoA derivatives involved in the biosynthesis of PECs was also suggested to be derived from the phenylpropanoid pathway (Figure 7). The plant shikimate pathway is the entry to the biosynthesis of phenylpropanoids and results in the biosynthesis of phenylalanine and tyrosine. The elimination of the amino group of phenylalanine and tyrosine is catalyzed by corresponding phenylalanine ammonia lyase (PAL) and tyrosine ammonia lyase (TAL), thus yielding cinnamic acid and coumaric acid, respectively [36]. Starting from them, numerous organic acids are produced by reduction of double bond or hydroxylation at different positions, and provide diverse aromatic CoA-esters to different biosynthetic pathways by the function of CoA ligase. Among them, 4-coumaroyl CoA is the most representative one which contributes to the biosynthesis of not only flavonoids but also other important polyketides such as stilbenes, lignin, and coumarins [36]. In the case of PECs, we suggest that cinnamoyl-CoA, 4-methoxycinnamoyl-CoA, 2-hydroxycinnamoyl-CoA, and feruloyl-CoA or their corresponding dihydro analogues serve as the C_6_–C_3_ carbon skeleton to act as the first starters and contribute to the construction of phenylethyl moiety of PECs. Detection of phenylpropionic acid, phenylpropionic acid methyl ester, and 4-methoxyphenylpropionic acid methyl ester by GC-MS analysis partly proved the above deduction. Although not as popular as 4-coumaroyl CoA, at least some of them, such as cinnamoyl-CoA [36], 4-methoxycinnamoyl-CoA [37,38], feruloyl-CoA [35], and 3-hydroxyphenylpropionyl-CoA [39], have been reported as substrates for type III PKSs in different plants. However, unlike the well-established biosynthesis of phenylpropanoid CoA thioesters, the formation of phenolic acids with a C_6_–C_1_ skeleton still remain obscure, and two possible pathways are proposed, either by a β-oxidative pathway as described for *Petunia hybrida* [40] or by an alternative nonoxidative pathway as identified in *Anthirrhinum majus* [41]. It was reported that several pathways for benzoic acid biosynthesis may coexist in a single plant. The β-oxidative pathway produced a C_6_–C_1_ skeleton from cinnamic acid acts firstly by the addition reaction between the double bond and a molecule of water to introduce hydroxy group, then by oxidation of the hydroxy into a carbonyl group, and finally by the elimination of the β-acetyl group by a reverse Claisen reaction to produce benzoyl CoA. Further reaction catalyzed by 2-hydroxylase upon benzoic acid produced 2-hydroxybenoic acid, which is also known as salicylic acid (SA). SA plays a very important role in plant defense and serves as a critical signal for the establishment of plant resistance against pathogen attack. On the basis of SA, a number of biologically relevant chemical modifications, including glucosylation, methylation, amino acid conjugation, and hydroxylation, produced diverse phenolic derivatives. Gentisic acid (GA), also named 2,5-dihydroxybenzoic acid, is the C5-hydroxylated derivative of SA, and it has been proposed to be a signal molecule for plant defense response in compatible, non-necrotizing interactions [42]. Therefore, SA and GA, as the plant-defense-related signals, may be related to the formation of PECs in the injured *Aquilaria* plants by contributing their corresponding CoA derivatives to the construction of chromones moiety of PECs.

It is known that the selection of substrates by different type III PKSs is one of the reasons for the production of diverse polyketides [43,44]. For example, CURS1~3 catalyzed the in vivo formation of curcuminoids which were isolated from *Curcuma longa* [44,45]. Then, in vitro analysis revealed that CURS2 preferred feruloyl-CoA as the starter and CURS3 preferred both feruloyl-CoA and *p*-coumaroyl-CoA, suggesting that the availability of different substrates and different expression levels of the CURS 1~3 involved in curcuminoid synthesis could account for the variety of curcuminoids in different cultivars of turmeric (*Curcuma longa*). It is possible that the PKSs catalyzed the formation of PECs in *Aquilaria* or other plants may have different substrate specificity, and therefore, result in the different compositions of PECs.

In general, 2-(2-phenylethyl)chromones, as important secondary metabolites in agarwood, are the product of complex physiological and biochemical reactions in injured *A. sinensis*. So far, the process of agarwood accumulation has not been fully understood, and the biological origin of PECs still remains unknown. In this study, dynamic changes of chemical constituents, especially 2-(2-phenylethyl)chromones, in agarwood samples collected in different periods were studied by GC-MS and UPLC-MS analysis. Based on the observed results, as well as by comparison and analysis of structural characteristics of reported PECs, the possible biosynthetic pathway of PECs, either of agarwood or nonagarwood origin, were elucidated by comparison with biosynthetic pathways of other structurally similar secondary metabolites such as curcuminoids and flavonoids. This is the first reports that fully discussed the biosynthesis of PECs, and it may serve as a basis, from the perspective of chemical constituents, for further studies on the mechanism of agarwood formation and discovery of biosynthetic genes of PECs.

## 3. Experimental

### 3.1. General

All the reference compounds were isolated and identified from agarwood in our previous study, and the purity was determined to be more than 98% by HPLC-UV analysis. The structures of 30 reference compounds are listed in Table 3. Acetonitrile and methanol of HPLC grade were purchased from Tedia (Fairfield, AL, USA). Chromatographic-grade absolute formic acid was purchased from ROE SCIENTIFIC INC (Shanxi, China). Ultrapure water was prepared with a Milli-Q Plus 185 purification system (Millipore, Bedford, MA, USA).

### 3.2. Material Treatment

The wood samples used in this study were collected during a fixed period in an *A.sinensis* plantation at Dingan Town, Haikou City, Hainan province, China. The agarwood inducer invented in our lab was used for stimulating the formation of resin in *A. sinensis* trees. Seven-year-old *A. sinensis* trees were chosen for the experiment of agarwood formation, and the experiment was carried out by us from 28 July 2014 to 28 July 2015. Voucher specimens were deposited at the Institute of Tropical Bioscience and Biotechnology, Chinese Academy of Tropical Agricultural Sciences.

To conduct the experiment, first of all, we needed to hold an electric drill at a 45-degree angle from the trunk and drill a hole at the depth of about 4 cm in the trunk above 45 cm off the ground. Then, 500 mL of prepared agarwood inducer was transfused into each tree. After that, the hole was blocked by rubber in case of rainwash and to prevent outflow of the inducer.

### 3.3. Sample Preparation

The trunk initially started to decay around the hole we drilled for the transfusion of agarwood inducer. A month later, we chose three trees and chopped off a wood block about 15-cm long, 10-cm wide, and 2-cm thick at the position around the hole for each tree. Then, rotten woods were collected from three trees in the planation every month within half a year, then at the 9th and the 12th month.

The dry woods were whittled into chips after the removal of whitewood, followed by subjecting them to ultrasonic extraction with ethyl ether for 30 min under the condition of ice-cold water. After another 3 min’ standing, the suspension was filtered. The extraction was performed totally three times, and ethyl ether extracts were obtained after vaporizing.

### 3.4. GC-MS Analysis

GC-MS analysis was performed with an Agilent gas chromatography instrument (GC 7820) equipped with a ZB-5MSI 5% Phenyl-95% Dimethylpolysiloxane column (30 m × 0.25 mm, 0.25 μm) and a mass selective detector (5977) (Agilent Technologies Co., Ltd., Santa Clara, CA, USA) with an ion trap detector in full scan mode under election impact ionization (70 eV). The carrier gas was helium, at a flow rate of 1.0 mL/min with split ratio of 50:1. 1 μL essential oil solution in methanol (HPLC grade) was injected into the front inlet of the gas chromatograph operating at 250 °C in the splitless mode. The operating parameters were that the oven program commenced at 50 °C (2 min) and increased at a rate of 5 °C/min to 310 °C where it was held for 10 min. The ion source temperature was set at 230 °C and the scan range was from 50 to 500 amu under full scan.

### 3.5. UPLC-MS Analysis

A Dionex UltiMate 3000 series HPLC system (Dionex Softron GmbH, Germering, Germany), equipped with a diode array detector, a vacuum degasser, a quaternary pump, and an autosampler, and electrospray ionization tandem mass spectrometry (Amazon SL, Bruker Daltonik GmbH, Bremen, Germany) were used for sample analysis. The data acquisition was supported by Bruker Compass Data Analysis 4.0 software. The separations were performed on a Dionex-Acclaim 120 C_18_ column (250 mm × 4.6 mm, 5 µm) at 26 °C. The mobile phase system was acetonitrile (A) and water with 0.5% acetic acid (B) at flow rate of 0.4 mL/min. The gradient elution program was as follows: 0–60 min, 25–55% A, 60–80 min, 55–80% A, 80–90 min, 80–100% A, and 90–95 min, 100% A. The sample injection volume for analysis was 20 µL. The detection wavelength was set at 254 nm for all the tested compounds. The ESI-MS analysis was acquired in the positive ion mode. Helium gas was used as the collision gas at the flow rate of 0.4 L/min and high-purity nitrogen gas was used as the nebulizer and drying gas at 6.0 L/min. The conditions for ESI-MS analysis were as follows: capillary voltage, −4000 V; end plate voltage, −500 V; drying gas temperature, 250 °C; and nebulizer pressure, 15 psi. The MS spectra were scanned from 70 to 2200 *m/z*. The MS/MS analysis was used to obtain the mass fractions of target ions. The extracts and standards were diluted in methanol to 1 mg/mL, respectively, and filtered through 0.45 µm membranes.

## Figures and Tables

**Table 1:** Volatile constituents of agarwood samples produced from the 1st to 12th months.

No.	Compounds				Molecular Formula	MW	Relative Content (%)							
							1	2	3	4	5	6	9	12
**1**	Nonanal				C_9_H_18_O	142			0.85	0.89	1.16	0.22		
**2**	4-Phenylbutan-2-one (Benzylacetone) ^△^				C_10_H_12_O	148		1.02	0.46	0.17	0.09	0.21	0.14	0.11
**3**	Phenylpropanoic acid methyl ester ^△^				C_10_H_12_O_2_	164								0.11
**4**	Phenylpropionic acid ^△^				C_9_H_10_O_2_	150		1.80	0.19	0.09		0.03		0.12
**5**	2,6-Di-*tert*-butyl-2,5-cyclohexadiene-1,4-dione				C_14_H_20_O_2_	220				0.04	0.12			
**6**	4-(4-Methoxyphenyl)butan-2-one (Anisylacetone) ^△^				C_11_H_14_O_2_	178		0.09	0.11	0.09	0.17	0.44	0.69	0.46
**7**	2,4- Di-*tert*—butylphenol ^△^				C_14_H_22_O	206	0.67	0.17	0.21	0.39	0.66	0.26	0.35	0.08
**8**	4-Methoxy-phenylpropanoic acid methyl ester ^△^				C_11_H_14_O_3_	194								0.07
**9**	(1*S*,3a*R*,4*S*,8a*S*)-4,8,8-trimethyl-9-methylenedecahydro-1,4-methanoazulene (Junipene) ^※^				C_15_H_24_	204				0.10				
**10**	2-[(3*R*,5*R*,6*R*)-6,10-dimethylspiro[4.5]dec-9-en-3-yl] propan-2-ol (Agarospirol) ^※^				C_15_H_26_O	222					0.68	0.09	0.88	0.02
**11**	2-[(1*R*,3*S*,4*S*)-4-ethenyl-4-methyl-3-prop-1-en-2-yl cyclohexyl]propan-2-ol (Elemol) ^※^				C_15_H_26_O	222				0.36				
**12**	2-[(2*R*,8*S*,8a*R*)-8,8a-dimethyl-2,3,4,4a,7,8-hexahydro-1*H*-naphthalen-2-yl]propan-2-ol [(-)-Jinkoh-eremol] ^※^				C_15_H_26_O	222				0.14	0.51	0.08	0.94	0.06
**13**	2-[(3*S*,5*R*,8*S*)-3,8-dimethyl-1,2,3,4,5,6,7,8-octahydroazulen-5-yl] propan-2-ol (Guaiol) ^※^				C_15_H_26_O	222						0.24	1.51	0.06
**14**	(1a*R*,4*S*,4a*S*,7*R*,7a*S*,7b*S*)-1,1,4,7-tetramethyl-octahydro-1a*H*-cyclopropa[e]azulen-4-ol (Viridiflorol) ^※^				C_15_H_26_O	222				0.16	0.81	0.50	0.45	0.05
**15**	2-Hydroxy-1,2,3-propanetricarboxylic acid triethyl ester (Triethyl citrate)				C_12_H_20_O_7_	276	1.59	0.69	1.01					
**16**	4-(4-Hydroxy-3-methoxyphenyl)butan-2-one (Zingerone) ^△^				C_11_H_14_O_3_	194							1.71	0.11
**17**	(1*S*,4*R*,5*S*,6*R*)-4,7,7-trimethylspiro[bicyclo[4.1.0]heptane-5,3′-cyclopentene]-1′-carbaldehyde (Vitrenal) ^※^				C_15_H_22_O	218					0.39	0.39		
**18**	3-(2-Hydroxypropan-2-yl)-6-methylspiro[4.5]dec-9-ene-10-carbaldehyde (Neopetasane) ^※^				C_15_H_22_O	218				0.22	0.34	0.36	0.58	0.26
**19**	3-(2-Hydroxypropan-2-yl)-6-methylspiro[4.5]dec-9-ene-10-carbaldehyde (Baimuxinal) ^※^				C_15_H_24_O_2_	236				2.45	1.41	1.91	7.46	1.63
**20**	Methyl hexadecanoate (Methyl palmitate) ^☆^				C_17_H_34_O_2_	270			0.58					0.47
**21**	1-*O*-butyl 2-*O*-(2-methylpropyl)benzene-1,2-dicarboxylate (Isobutyl phthalate) ^△^				C_16_H_23_O_4_	278	0.47	0.08	0.47		0.38		0.51	
**22**	Hexadecanoic acid (Palmitic acid) ^☆^				C_16_H_32_O_2_	256		0.27	0.27	1.85		3.27		2.59
**23**	Propan-2-yl hexadecanoate (isopropyl palmitate) ^☆^				C_19_H_38_O_2_	298					1.94			
**24**	Methyl octadecanoate (Methyl stearate) ^☆^				C_19_H_38_O_2_	298			0.33					0.06
**25**	(*Z*)-octadec-9-enoic acid (Oleic acid) ^☆^				C_18_H_34_O_2_	282				0.62		6.50		0.65
**26**	1,5-Diphenyl-1-penten-3-one ^△^				C_17_H_16_O	236		0.17			0.51			
**27**	Methyl *cis*-9,10-epoxystearate ^☆^				C_19_H_36_O_3_	312	0.66		0.65					
**28**	2-(2-Phenylethyl)chromone *				C_17_H_14_O_2_	250	8.71	20.34	6.31	8.91	6.49	1.18	0.48	2.36
**29**	Bis(6-methylheptyl) benzene-1,2-dicarboxylate (Diisooctyl phthalate) ^△^				C_24_H_38_O_4_	390	0.85	0.16	0.46	0.22	0.50	1.40	0.45	0.92
**30**	5 or 6 or 7 or 8-Hydroxy-2-(2-phenylethyl)chromone *				C_17_H_14_O_3_	266		1.02	0.94	0.59	0.39	0.40		0.22
**31**	5 or 6 or 7 or 8-Methoxy-2-(2-phenylethyl)chromone *				C_18_H_16_O_3_	280	12.63	14.76	8.13	14.40	8.69	4.55	5.72	12.69
**32**	2-[2-(4-Methoxyphenyl)ethyl]chromone *				C_18_H_16_O_3_	280	Overlapped with No. 31							
**33**	5 or 6 or 7 or 8-Methoxy-2-(2-phenylethyl)chromone *				C_18_H_16_O_3_	280	0.95	0.45	0.47	0.56	0.43	0.16	0.35	
**34**	2-(2-Phenylethyl)chromone: A ring: 1OH, 1OCH_3_ *				C_18_H_16_O_4_	296	0.95	1.27	1.26	1.73	0.80	0.64	1.01	2.40
**35**	6,8-Dihydroxy-2-(2-phenylethyl)chromone *				C_17_H_14_O_4_	282	1.22	4.48	2.55	1.83	1.13	1.04	1.44	1.73
**36**	5 or 6 or 7 or 8-Hydroxy-2-(2-phenylethyl)chromone *				C_17_H_14_O_3_	266	9.28	21.60	12.10	8.09	4.25	5.20	3.03	6.13
**37**	2-(2-Phenylethyl)chromone: A ring: 1OH, 1OCH_3_ *				C_18_H_16_O_4_	296			0.81	1.01	0.88	0.92	0.36	1.56
**38**	6-Methoxy-2-[2-(3-methoxyphenyl)ethyl]chromone *				C_19_H_18_O_4_	310	4.15	3.35	1.84	3.29	3.34	2.80	1.85	2.25
**39**	6,7-Dimethoxy-2-(2-phenylethyl)chromone *				C_19_H_18_O_4_	310	44.25	18.15	22.12	24.12	17.99	13.99	18.64	24.87
**40**	5,8-Dihydroxy-2-[2-(4-methoxyphenyl)ethyl]chromone *				C_18_H_16_O_5_	312		0.43	0.60	0.57		7.37	0.82	2.73
**41**	2-(2-Phenylethyl)chromone: A ring: 1OH, 1OCH_3_ *				C_18_H_16_O_4_	296		1.41	1.46	1.36	1.06	2.03	0.96	1.59
**42**	6-Hydroxy-2-[2-(4-methoxyphenyl)ethyl]chromone *				C_18_H_16_O_4_	296		0.95	2.18	1.59	0.45	4.08	1.90	2.10
**43**	6,7-Dimethoxy-2-[2-(4-methoxyphenyl)ethyl]chromone *				C_20_H_20_O_5_	340	1.74	1.26	2.97	2.15	2.24	3.96	5.63	3.96
**44**	Ergost-5-enol ^○^				C_28_H4_8_O	400		0.05	0.36	0.12				0.30
**45**	Stigmasterol ^○^				C_29_H_48_O	412	2.62	0.92	3.48	1.66	1.96	1.27	3.52	5.03
**46**	Clionasterol ^○^				C_29_H_50_O	414	1.76	0.30	2.80	1.23	1.50	1.75	2.42	2.60
**47**	5α-Stigmast-3-one ^○^				C_29_H_50_O	414		0.05	0.28	0.11				
**48**	Ergosta-4,6,8(14),22-tetraen-3-one ^○^				C_28_H_40_O	392			0.80	0.25	0.81			
**49**	(22*E*,24*R*)-Stigmasta-4,22-dien-3-one ^○^				C_29_H_46_O	410		0.06	0.58	0.20	2.26			
**50**	Stigmast-4-en-3-one ^○^				C_29_H_48_O	412	2.99	0.53	1.89	1.41	8.27	1.93	2.21	0.96
**51**	Stigmasta-3,5-dien-7-one ^○^				C_29_H_46_O	410					0.31		0.90	
**52**	(5α)-Stigmastane-3,6-dione ^○^				C_29_H_48_O_2_	428	0.81	0.32	1.55	0.79	4.41		0.98	0.45
		**1**	**2**	**3**	**4**		**5**		**6**		**9**		**12**	
	Aromatic compounds (No./RC, %)	3/ 1.99	7/ 3.50	7/2.74	6/1.86		7/3.47		6/2.55		7/3.98		8/1.92	
	Fatty acids/esters (No./RC, % )	2/2.24	2/0.97	4/2.20	2/2.47		1/1.94		2/9.76		\		4/3.77	
	Steroids (No./RC, %)	4/8.18	7/2.23	8/11.74	8/5.78		7/19.51		3/4.95		6/10.93		5/9.34	
	Sesquiterpenoids (No./RC, %)	\	\	\	7/3.74		6/4.14		7/3.57		6/11.38		6/2.14	
	2-(2-phenylethyl)chromones (No./RC, %)	10/83.86	13/89.46	15/64.32	15/70.78		14/48.14		15/49.74		14/42.19		13/64.59	
	Identified compounds (No./RC, %)	19/96.28	30/96.16	35/81.00	39/84.62		35/77.21		33/70.58		33/68.48		36/81.76	

Note: MW = Molecular Weight, * 2-(2-phenylethyl)chromones, ^○^ Steroids, ^△^ Aromatic compounds, ^☆^ Fatty acids/esters, ^※^ Sesquiterpenoids. RC is the relative content.

**Table 2:** The UPLC-MS analysis results of agarwood samples produced from the 1st to 12th months.

No	Compounds			Molecular Formula	MW	Relative Content (%)							
						1	2	3	4	5	6	9	12
**1**	THPECs: A ring: 4 OH groups, C ring: 1 OH, 1 OCH_3_			C_18_H_20_O_8_	364					2.27	7.89	5.78	3.11
**2**	S1: 5α,6α,7α,8β-Tetrahydroxy-2-[2-(3-hydroxy-4-methoxyphenyl) ethyl]-5,6,7,8-tetarahydrochromone			C_18_H_20_O_8_	364							1.43	
**3**	S2: 5α,6β,7β,8α-Tetrahydroxy-2-[2-(3-hydroxy-4-methoxyphenyl) ethyl]-5,6,7,8-tetarahydrochromone			C_18_H_20_O_8_	364					0.78	1.57	2.48	0.22
**4**	S3: 5α,6β,7β,8α-Tetrahydroxy-2-[2-(4-methoxyphenyl)ethyl]-5,6,7,8-tetrahydrochromone			C_18_H_20_O_7_	348	8.27	9.13	7.47	10.56	8.26	14.73	6.05	5.11
**5**	S4: 5α,6β,7β,8α-Tetrahydroxy-2-[2-(4-methoxyphenyl)ethyl)]-5,6,7,8-tetrahydrochromone (Agaroretrol)			C_17_H_18_O_6_	318	10.00	0.99	5.08	0.88	0.46	1.35	0.38	0.43
**6**	THPECs: A ring: 4 OH groups, C ring: 1 OCH_3_			C_18_H_20_O_7_	348								0.10
**7**	THPECs: A ring: 4 OH groups			C_17_H_18_O_6_	318	0.41	0.24	0.82	0.85	0.99	1.61	1.50	0.22
**8**	THPECs: A ring: 4 OH groups			C_17_H_18_O_6_	318	1.79	4.65	2.60	4.65	4.63	6.90	2.70	3.71
**9**	S5: 5:6-Epoxy-7β,8α-dihydroxy-2-[2-(3-hydroxy-4-methoxyphenyl) ethyl]-5,6,7,8-tetarahydrochromone			C_18_H_18_O_7_	346					0.13	0.08	1.21	0.80
**10**	THPECs: A ring: 3 OCH_3_, 1 OH			C_20_H_24_O_6_	360	0.07	0.28		0.20				
**11**	THPECs: A ring: 3 OCH_3_, 1 OH, C ring: 1 OCH_3_			C_18_H_20_O_8_	390								0.26
**12**	THPECs: A ring: 3 OH, 1 OCH_3_			C_18_H_20_O_6_	332								0.11
**13**	S6: (5*R*,6*R*,7*R*,8*R*)-5,6 :7,8-Diepoxy-2-[2-(3-hydroxy-4-methoxyphenyl) ethyl]-5,6,7,8-tetrahydrochromone (Oxidoagarochromone C)			C_18_H_16_O_6_	328					0.07	0.87	1.25	0.02
**14**	DEPECs: C ring: 1 OCH_3_			C_18_H_16_O_5_	312					0.05		0.45	1.17
**15**	EPECs: 7′-OH			C_17_H_14_O_4_	282								1.15
**16**	DEPECs: C ring: 1 OCH_3_, 1 OH			C_18_H_16_O_6_	328					0.35	1.21	1.30	0.22
**17**	THPECs: A ring: 3 OH groups, 1 Cl, C ring: 1 OCH_3_			C_18_H_19_O_6_Cl	366								0.30
**18**	THPECs: A ring: 3 OH groups, 1 Cl			C_17_H_17_O_5_Cl	336	1.28	0.16	1.08	0.12		0.51		0.19
**19**	S7: 6-Hydroxy-7-methoxy-2-[2-(4-hydroxyphenyl)ethyl]chromone			C_18_H_16_O_5_	312							0.46	
**20**	FTPECs: A ring: 1 OH, 1 OCH_3_, C ring: 1 OH			C_18_H_16_O_5_	312							0.38	0.04
**21**	FTPECs: A ring: 1 OH, 1 OCH_3_, C ring: 1 OH, 1 OCH_3_			C_19_H_18_O_6_	342								0.09
**22**	S8: 5:6-Epoxy-7β,8α-dihydroxy-2-[2-(4-methoxyphenyl)ethyl]chromone			C_18_H_18_O_6_	330		0.05	0.12	0.20				2.80
**23**	EPECs: A ring: 2 OH groups, C ring: 1 OCH_3_			C_18_H_18_O_6_	330			0.18	0.16				
**24**	FTPECs: A ring 1 OH, 1 OCH_3_, C ring: 1 OH, 1 OCH_3_			C_19_H_18_O_6_	342					0.85	0.95	2.24	1.60
**25**	S9: 7-hydroxy-6-methoxy2-[2-(3-hydroxy-4-methoxyphenyl)ethyl] chromone			C_19_H_18_O_6_	342					0.42	0.38	0.93	0.67
**26**	S10: 5:6-Epoxy-7β,8α-dihydroxy-2-(2-phenylethyl)chromone			C_17_H_16_O_5_	300	1.57	2.12	1.52	0.65				1.12
**27**	THPECs: A ring: 3 OH groups, 1 Cl			C_17_H_17_O_5_Cl	336	0.14	0.15	0.37	0.34				
**28**	FTPECs: A ring: 1 OH, C ring: 1 OH, 1 OCH_3_			C_18_H_16_O_5_	312					1.35	1.57	2.55	1.67
**29**	DEPECs: C ring: 1 OCH_3_			C_18_H_16_O_5_	312								0.07
**30**	S11: 6-Hydroxy-2-[2-(3-hydroxy-4-methoxyphenyl)ethyl]chromone			C_18_H_16_O_5_	312					0.50	0.48	0.51	0.40
**31**	S12: 6,8-Dihydroxy-2-[2-(4-methoxyphenyl)ethyl]chromone			C_18_H_16_O_5_	312				0.60	0.20	1.13	0.56	1.00
**32**	S13: 6,8-Dihydroxy-2-(phenylethyl)chromone			C_17_H_14_O_4_	282	1.23	2.78	1.50	1.28		1.03	0.54	0.86
**33**	S14: 6,7-Dimethoxy-2-[2-(4-hydroxyphenyl)ethyl]chromone			C_19_H_18_O_5_	326							0.37	0.51
**34**	S15: 6-Hydroxy-2-[2-(2-hydroxyphenyl)ethyl]chromone			C_17_H_14_O_4_	282			0.24	0.27				0.02
**35**	S16: (5*R*,6*R*,7*R*,8*R*)-5,6 :7,8-Diepoxy-2-[2-(4-methoxyphenyl)ethyl]-5,6,7,8-tetrahydrochromone (Oxidoagarochromone B)			C_18_H_16_O_5_	312	0.16	0.14	0.76	1.06	1.87	1.81	0.39	1.01
**36**	S17: (5*R*,6*R*,7*R*,8*R*)-5,6 :7,8-Diepoxy-2-(2-phenylethyl)-5,6,7,8-tetrahydrochromone (Oxidoagarochromone A)			C_17_H_14_O_4_	282	5.08							
**37**	S18: 6,7-Dimethoxy-2-[2-(3-hydroxy-4-methoxyphenyl)ethyl]chromone			C_20_H_20_O_6_	356							0.47	1.33
**38**	FTPECs: A ring: 2 OH groups			C_17_H_14_O_4_	282	0.20	3.45	1.74	2.29	1.08	1.76	0.98	0.67
**39**	FTPECs: C ring: 1 OH			C_17_H_14_O_3_	266			0.04	0.33	0.13	0.15	0.28	0.07
**40**	S19: 2-[2-(4-Hydroxy-3-methoxyphenyl)ethyl]chromone			C_18_H_16_O_4_	296							0.43	0.32
**41**	FTPECs: A ring: 2 OCH_3_ groups, C ring: 1 OH			C_19_H_18_O_5_	326	0.96	0.25	0.39					
**42**	S20: 8-Hydroxy-2-(2-phenylethyl)chromone			C_17_H_14_O_3_	266	1.02	0.88	1.07	1.06	0.59	0.61	0.80	0.65
**43**	FTPECs: A ring: 1 OH, 1 OCH_3_, C ring: 1 OCH_3_			C_19_H_18_O_5_	326	0.15	0.07	0.75	0.38	0.86	0.92	1.31	2.02
**44**	S21: 7-Hydroxy-6-methoxy2-[2-(4-methoxyphenyl)ethyl]chromone			C_19_H_18_O_5_	326				0.32	0.73	0.64	0.73	0.20
**45**	FTPECs: A ring: 1 OH, 1 OCH_3_			C_18_H_16_O_4_	296	1.97	1.41	2.33	1.17	1.75	1.57	1.88	2.46
**46**	7-hydroxy-6-methoxy2-(2-phenylethyl)chromone			C_18_H_16_O_4_	296	0.86	0.50	1.09	1.24	1.38	1.12	0.79	0.42
**47**	S22: 2-[2-(2-Hydroxyphenyl)ethyl]chromone			C_17_H_14_O_3_	266	0.74	0.35	0.47	0.17				
**48**	S23: 6-Methoxy-2-[2-(3-hydroxy-4-methoxyphenyl)ethyl]chromone			C_19_H_18_O_5_	326					0.06		0.77	0.11
**49**	S24: 6-Hydroxy-2-[2-(4-methoxyphenyl)ethyl]chromone			C_18_H_16_O_4_	296	0.52	1.06	3.58	2.57	7.90	4.46	2.56	5.81
**50**	S25: 6-Hydroxy-2-(2-phenylethyl)chromone			C_17_H_14_O_3_	266	14.07	19.15	15.60	12.12	14.96	6.86	2.96	10.55
**51**	S26: 5-Hydroxy-6-methoxy-2-[2-(3-hydroxy-4-methoxyphenyl)ethyl]chromone			C_19_H_18_O_6_	342						0.25	0.45	0.14
**52**	FTPECs: A ring: 1 OH, C ring: 1OH			C_17_H_14_O_4_	282		0.67	0.10	0.27				0.09
**53**	6,7-Dimethoxy-2-[2-(4-methoxyphenyl)ethyl]chromone			C_20_H_20_O_5_	340	2.19	1.10	3.32	2.67	4.65	6.00	5.58	4.30
**54**	S27: 6,7-Dimethoxy-2-(2-phenylethyl)chromone			C_19_H_18_O_4_	310	23.59	12.74	18.94	17.75	15.06	14.25	17.54	18.56
**55**	FTPECs: A ring: 2 OCH_3_ groups			C_19_H_18_O_4_	310			0.20	0.25		0.43	3.29	
**56**	FTPECs: A ring: 2 OH, C ring: 1 OCH_3_			C_18_H_16_O_5_	312					1.31			0.44
**57**	FTPECs: A ring: 2 OH groups			C_17_H_14_O_4_	282		1.21	0.80	1.17				1.36
**58**	S28: 2-[2-(4-Methoxyphenyl)ethyl]chromone			C_18_H_16_O_3_	280	0.27	0.39	0.53	0.38	1.60	0.51	0.46	0.62
**59**	S29: 2-(2-Phenylethyl)chromone			C_17_H_14_O_2_	250	5.83	13.55	4.74	5.18	5.53	1.90	0.81	2.33
**60**	6-Methoxy-2-[2-(4-methoxyphenyl)ethyl]chromone			C_19_H_18_O_4_	310	2.96	2.90	2.21	2.92	3.62	2.56	1.74	3.02
**61**	6-Methoxy-2-(2-phenylethyl)chromone			C_18_H_14_O_3_	280	6.91	10.60	6.54	19.12	8.75	4.87	5.00	9.89
**62**	FTPECs: A ring: 2 OCH_3_ groups, 1 OH, C ring: 1 OCH_3_			C_20_H_20_O_6_	356						0.24	0.59	0.28
**63**	S30: 5-Hydroxy-6-methoxy-2-[2-(4-methoxyphenyl)ethyl]chromone			C_19_H_18_O_5_	326	1.13	0.90	0.70	0.73	0.87	1.06	1.06	0.86
**64**	5-Hydroxy-6-methoxy-2-(2-phenylethyl)chromone			C_18_H_16_O_4_	296	2.86	2.50	2.96	3.38	2.34	2.44	3.68	3.13
	**1**	**2**	**3**	**4**		**5**		**6**		**9**		**12**	
FTPECs (n/RC, %)	18/67.46	20/76.46	23/69.84	24/77.62		24/76.49		26/58.14		32/62.70		35/75.59	
THPECs (n/RC, %)	7/21.96	7/15.60	6/17.42	7/17.60		6/17.39		7/34.56		7/20.32		11/13.76	
EPECs (n/RC, %)	1/1.57	2/2.17	4/3.34	4/1.66		1/0.13		1/0.08		1/1.21		4/5.87	
DEPECs (n/RC, %)	2/5.24	1/0.14	1/0.76	1/1.06		4/2.34		3/3.89		4/4.21		5/2.49	
Total (n/RC, %)	28/96.23	30/94.37	34/91.36	36/97.94		35/96.35		37/96.67		44/88.44		55/97.71	

**Table 3:** Characterization of 30 reference compounds by using UPLC/ESI-MS/MS.

No.	Structure	t_R_ (min)	[M + H]^+^ (*m*/*z*)	Fragment Ions (*m*/*z*)	Name
S1	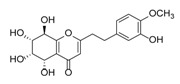	9.6	365	347, 329	5α,6α,7α,8β-Tetrahydroxy-2-[2-(3-hydroxy-4-methoxyphenyl)ethyl]-5,6,7,8-tetarahydrochromone
S2	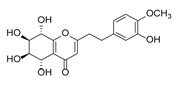	9.9	365	347, 329, 301	5α,6β,7β,8α-Tetrahydroxy-2-[2-(3-hydroxy-4-methoxyphenyl)ethyl]-5,6,7,8-tetarahydrochromone
S3	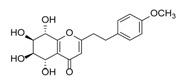	13.0	349	331, 137	5α,6β,7β,8α-Tetrahydroxy-2-[2-(4-methoxyphenyl)ethyl]-5,6,7,8-tetarahydrochromone
S4	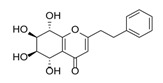	14.1	319	301, 283	5α,6β,7β,8α-Tetrahydroxy-2-[2-(4-methoxyphenyl)ethyl)]-5,6,7,8-tetrahydrochromone (Agaroretrol)
S5	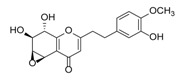	18.4	347	329, 301	5:6-Epoxy-7β,8α-dihydroxy-2-[2-(3-hydroxy-4-methoxyphenyl)ethyl]-5,6,7,8-tetarahydrochromone
S6	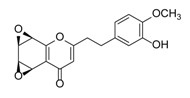	23.4	329	301, 137	(5*R*,6*R*,7*R*,8*R*)-5,6 :7,8-Diepoxy-2-[2-(3-hydroxy-4-methoxyphenyl)ethyl]-5,6,7,8-tetrahydrochromone (Oxidoagarochromone C)
S7	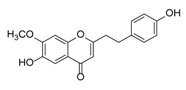	30.0	313	207, 107	6-Hydroxy-7-methoxy-2-[2-(4-hydroxyphenyl)ethyl]chromone
S8	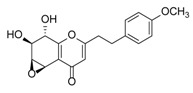	32.7	331	313, 121	5:6-Epoxy-7β,8α-dihydroxy-2-[2-(4-methoxyphenyl) ethyl]chromone
S9	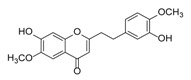	32.8	343	137	6-Methoxy-7-hydroxy-2-[2-(3-hydroxy-4-methoxyphenyl) ethyl]chromone
S10	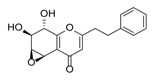	33.4	301	283, 255	5:6-Epoxy-7β,8α-dihydroxy-2-(2-phenylethyl)chromone
S11	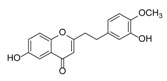	36.6	313	137	6-Hydroxy-2-[2-(3-hydroxy-4-methoxyphenyl)ethyl]chromone
S12	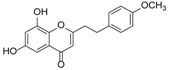	38.2	313	121	6,8-Dihydroxy-2-[2-(4-methoxyphneyl)ethyl]chromone
S13	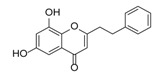	39.3	283	192, 91	6,8-Dihydroxy-2-(phenylethyl)chromone
S14	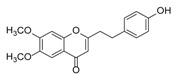	40.5	327	221, 107	6,7-Dimethoxy-2-[2-(4-hydroxyphenyl)ethyl]chromone
S15	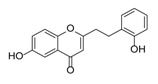	41.0	283	177, 107	6-Hydroxy-2-[2-(2-hydroxyphenyl)ethyl]chromone
S16	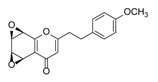	42.3	313	285, 121	(5*R*,6*R*,7*R*,8*R*)-5,6 :7,8-Diepoxy-2-[2-(4-methoxyphenyl)ethyl]-5,6,7,8-tetrahydrochromone (Oxidoagarochromone B)
S17	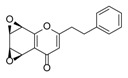	42.7	283	255	(5*R*,6*R*,7*R*,8*R*)-5,6 :7,8-Diepoxy-2-(2-phenylethyl)-5,6,7,8-tetrahydrochromone (Oxidoagarochromone A)
S18	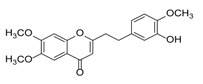	42.9	357	137	6,7-Dimethoxy-2-[2-(3-hydroxy-4-methoxyphenyl)ethyl] chromone
S19	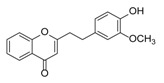	47.7	297	161, 137	2-[2-(4-hydroxy 3-methoxyphenyl)ethyl]chromone
S20	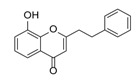	49.2	267	91, 176	8-Hydroxy-2-(2-phenylethyl)chromone
S21	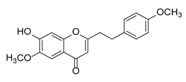	51.4	327	121	7-Hydroxy-6-methoxy2-[2-(4-methoxyphenyl)ethyl]chromone
S22	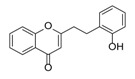	54.1	267	161	2-[2-(2-Hydroxyphenyl)ethyl]chromone
S23	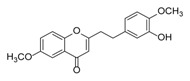	54.4	327	137	6-Methoxy-2-[2-(3-hydroxy-4-methoxyphenyl)ethyl]chromone
S24	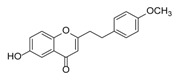	55.6	297	121	6-Hydroxy-2-[2-(4-methoxyphenyl)ethyl]chromone
S25	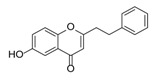	57.3	267	91	6-Hydroxy-2-(2-phenylethyl)chromone
S26	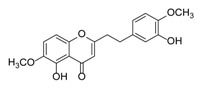	58.8	343	137	5-Hydroxy-6-methoxy-2-[2-(3-hydroxy-4-methoxyphenyl)ethyl]chromone
S27	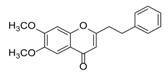	64.4	311	220	6,7-Dimethoxy-2-(2-phenylethyl)chromone
S28	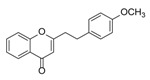	71.2	281	121	2-[2-(4-Methoxyphenyl)ethyl]chromone
S29	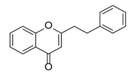	73.0	251	160, 91	2-(2-Phenylethyl)chromone
S30	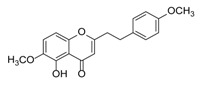	78.4	327	206, 121	5-Hydroxy-6-methoxy-2-[2-(4-methoxyphenyl)ethyl]chromone

**Table 4:** Summarizing PECs of nonagarwood origin.

No.	Coumpund	Resources	Literature
I	2-(2-phenylethyl)chromone (flindersiachromone)	*Flindersia laevicarpa* (Rutaceae) *Imperata cylindrica* (Gramineae)	[21] [23,25]
II	5-hydroxy-6-methoxy-2-[2-(2-hydroxyphenyl)-ethyl]-chromone	*Bothriochloa ischaemum* (Gramineae)	[22]
III	2,3-dihydro-2-(2-phenylethyl)chromone-3-one	*Flindersia laevicarpa* (Rutaceae)	[21]
IV	1,5-diphenylpentane-1,3-diol	*Flindersia laevicarpa* (Rutaceae)	[21]
V	5,7-dihydroxy-2-[2-(4-hydroxyphenyl)ethyl]chromone	*Cucumis melo* (Cucurbitaceae)	[24]
VI	5,7-dihydroxy-2-[2-(3,4-dihydroxyphenyl)ethyl]chromone	*Cucumis melo* (Cucurbitaceae)	[24]
VII	5,7-dihydroxy-2-[2-(3-methoxy-4-hydroxyphenyl)ethyl]chromone	*Cucumis melo* (Cucurbitaceae)	[26]
VIII	7-glucosyloxy-5-hydroxy-2-[2-(4-hydroxyphenyl)ethyl]chromone	*Cucumis melo* (Cucurbitaceae)	[24]
IX	5-hydroxy-2-(2-phenylethyl)chromone	*Imperata cylindrica* (Gramineae)	[23]
X	5-hydroxy-2-[2-(2-hydroxypheny1)-ethyl]-chromone	*Bothriochloa ischaemum* (Gramineae) *Imperata cylindrica* (Gramineae)	[22] [23]
XI	5-hydroxy-2-styrylchromone	*Imperata cylindrica* (Gramineae)	[23]
XII	8-hydroxy-2-(2-phenylethyl)chromone	*Imperata cylindrica* (Gramineae)	[25]
XIII	8-methoxy-flindersiachromone	*Flindersia laevicarpa* (Rutaceae)	[21]
XIV	2-(2-phenylethyl)chromone-8-*O*-β-d-glucopyranoside	*Imperata cylindrica* (Gramineae)	[25]

**Table 5:** The statistic results of the total relative content (%) from the 1st to 12th month of three groups of 2-(2-phenylethyl)chromones which were subdivided according to the substitution pattern of phenylethyl moiety.

	1	2	3	4	5	6	9	12
Group І	77.63	75.56	66.53	71.62	57.52	46.27	39.56	55.0
Group II	15.50	15.67	18.69	22.01	29.70	32.90	19.13	24.73
Group Ш	\	\	\	\	1.96	3.63	9.93	3.69
